# Incorporating Wearable Technology for Enhanced Rehabilitation Monitoring after Hip and Knee Replacement

**DOI:** 10.3390/s24041163

**Published:** 2024-02-10

**Authors:** Julien Lebleu, Kim Daniels, Andries Pauwels, Lucie Dekimpe, Jean Mapinduzi, Hervé Poilvache, Bruno Bonnechère

**Affiliations:** 1 moveUp, 1000 Brussels, Belgium; julien@moveup.care (J.L.); andries@moveup.care (A.P.); lucie@moveup.care (L.D.); 2 Department of PXL—Healthcare, PXL University of Applied Sciences and Arts, 3500 Hasselt, Belgium; kim.daniels@pxl.be; 3 REVAL Rehabilitation Research Center, Faculty of Rehabilitation Sciences, Hasselt University, 3590 Diepenbeek, Belgium; mapinduzi.jean@yahoo.fr; 4 Filière de Kinésithérapie et Réadaptation, Département des Sciences Clinique, Institut National de la Santé Publique, 6807 Bujumbura, Burundi; 5 Orthopedic Surgery Department, CHIREC, 1420 Braine-l’Alleud, Belgium; 6 Technology-Supported and Data-Driven Rehabilitation, Data Sciences Institute, Hasselt University, 3590 Diepenbeek, Belgium

**Keywords:** wearable sensors, activity tracker, mHealth, osteoarthritis, rehabilomics, rehabilitation, personalized care

## Abstract

Osteoarthritis (OA) poses a growing challenge for the aging population, especially in the hip and knee joints, contributing significantly to disability and societal costs. Exploring the integration of wearable technology, this study addresses the limitations of traditional rehabilitation assessments in capturing real-world experiences and dynamic variations. Specifically, it focuses on continuously monitoring physical activity in hip and knee OA patients using automated unsupervised evaluations within the rehabilitation process. We analyzed data from 1144 patients who used a mobile health application after surgery; the activity data were collected using the Garmin Vivofit 4. Several parameters, such as the total number of steps per day, the peak 6-minute consecutive cadence (P6MC) and peak 1-minute cadence (P1M), were computed and analyzed on a daily basis. The results indicated that cadence-based measurements can effectively, and earlier, differ among patients with hip and knee conditions, as well as in the recovery process. Comparisons based on recovery status and type of surgery reveal distinctive trajectories, emphasizing the effectiveness of P6MC and P1M in detecting variations earlier than total steps per day. Furthermore, cadence-based measurements showed a lower inter-day variability (40%) compared to the total number of steps per day (80%). Automated assessments, including P1M and P6MC, offer nuanced insights into the patients’ dynamic activity profiles.

## 1. Introduction

Osteoarthritis (OA) is a prominent contributor to disability and a significant driver of societal expenditure among the older population. The prevalence of this condition is on the rise due to a combination of factors, including an aging population and a growing prevalence of obesity, trauma, abnormal joint morphology, developmental dysplasia of the joint, muscle weakness of the joint, occupation (high-impact physical activity or sport), sedentarity, etc. [1,2].

The hip and knee joints, which bear the weight of the body, are the most prominently affected [3]. Consequently, they stand as the primary drivers of disability among the older population. The prevalence of OA shows a consistent upward trajectory with advancing age, affecting approximately 20% of individuals aged 60 or older [4].

The rehabilitation process holds significant importance for patients who have been diagnosed with hip and knee OA following total hip or knee replacement [5,6]. Historically, the examination and management of these individuals have predominantly relied on supervised clinical assessments conducted within controlled clinical environments [7]. Nevertheless, it is important to acknowledge that these assessments possess inherent limits in their ability to fully capture the intricate nature of real-world experiences encountered during the process of rehabilitation [8]. Furthermore, traditional methods evaluate data at a specific moment, assuming the stability of parameters over time. However, the intricate dynamics of real-life scenarios encompass physical and temporal variations throughout the day, week, and so forth, particularly in the individual-level factors such as pain, stress, emotion, and motivation.

The use of technology-assisted rehabilitation is gaining popularity due to its ability to provide objective and automated assessments of patients’ motor function and therapy adherence, incorporating factors like kinematics, activity level, muscle activity, and more [9]. Mobile health technologies, including wearable and portable sensors, are now being used to assess mobility in unsupervised, real-world situations, offering a more patient-relevant and ecologically valid approach compared to routine clinical tests [10]. This approach helps overcome the limitations of traditional clinical assessments where the outcomes used in the research study, mainly subjective evaluated by the clinicians, may not be sensitive enough to detect subtle modifications in the patients because of low resolution and the ceiling effect [11]. Quantitative outcomes used to validate the intervention are, most of the time, based on clinical or laboratory assessment, which are both quite artificial situations not reflecting the activities of daily living and the real-world evidence [10].

To address this research gap, the present study focuses on leveraging wearable technology, particularly Garmin activity trackers, to provide continuous and objective monitoring of physical activity in hip and knee OA patients undergoing total hip arthroplasty (THA) and total knee arthroplasty (TKA).

The implementation of automated unsupervised evaluations signifies a substantial shift in the approach to evaluating and providing support for patients during their rehabilitation process. The potential advantages are diverse, encompassing enhanced precision in assessments; the possibility of tailored, patient-centered therapies; and heightened patient involvement in their own rehabilitation journey [12,13]. The objective of this study was to examine the potential of automated technology in augmenting the capacities of patients and rehabilitation specialists.

Remote health monitoring, facilitated by non-invasive wearable sensors and modern communication technologies, ensures patient safety at home while enabling continuous data collection between sessions [14]. These data can detect even minor changes in a patient’s status, providing more precise and sensitive outcomes, known as digital biomarkers [15]. Digital biomarkers, collected through various sensors such as accelerometers, smartwatches, connected insoles, and smartphones, offer continuous and objective measurements of biological and physiological data [16]. They can reveal disease characteristics not easily observable in clinical settings. Digital biomarkers can be categorized as active (supervised) or passive (unsupervised) [17].

The widespread adoption of smartphones equipped with built-in accelerometers and gait-detection algorithms has introduced a higher level of detail in monitoring mobility data [18]. Recent research efforts have applied accelerometry techniques to individuals undergoing joint arthroplasty [15,19]. Given the positive impact of physical activity on enhancing functionality, promoting bone healing, and ensuring the stability of implants, the focus of these studies has been on tracking step count recovery [20]. This assessment aims to determine if post-operative activity levels surpass those recorded before the surgery and the duration required to achieve this [21].

This method capitalizes on smartphones’ ability to capture activity data in real-world conditions, spanning a diverse range of activities [22]. However, it is crucial to recognize that these assessments are susceptible to variations influenced by how subjects carry their phones [23]. Owing to technological advancements and cost reductions, the popularity of smartwatches and activity trackers has been on the rise not only among the general population but also for medical purposes [24]. The past research has delved into the utilization of activity trackers to assess the physical activity levels of individuals undergoing hip and knee arthroplasty, as well as those with osteoarthritis. The findings indicate that arthroplasty is linked to elevated levels of physical activity compared to participants in the end-stage arthritis phase [25].

Encouragingly, the self-administered six-minute walk test (6MWT) has exhibited satisfactory accuracy, reproducibility, and acceptability in both healthy individuals and those with varying degrees of congestive heart failure severity [26]. Nevertheless, the exploration of unintentional walk testing, a method analyzing free-living physical activity data, remains an unexplored area in the current research, despite its potential to more accurately portray daily functional status. It has, for example, shown that individuals in the advanced stages of knee OA exhibit diminished physical function, characterized by a reduced intensity in walking [27]. The existing literature primarily focuses on daily step counts and sedentary time, offering limited insights into stepping bouts or cadence patterns. The exploration of associations between prolonged durations of higher stepping cadence on a daily basis and clinical outcomes represents a crucial step in advancing the understanding of optimal daily stepping recommendations in this context.

Therefore, in the present study, we investigated the use of activity tracker on quantifying patient activities of daily living and follow-up. The primary objective of this study was to examine the incorporation of unsupervised evaluations within the rehabilitation process in order to obtain a more multidimensional evaluation of the patient’s progression throughout their recovery trajectory, taking into account the dynamic obstacles they encounter throughout their reintegration into their regular routines.

This study proposes the incorporation of automated unsupervised assessments as an essential element in the rehabilitation process for persons after hip and knee replacement. The development of this method is rooted in the need for a more patient-centered, ecologically sound approach to rehabilitation assessments. By automatically collecting step-per-minute data and extracting key parameters such as cadence, the study aims to offer a nuanced understanding of patients’ dynamic activity profiles during the rehabilitation process.

## 2. Materials and Methods

### 2.1. Data Source

We conducted a retrospective observational study using anonymized depersonalized data. A cohort of 1144 patients who underwent elective total knee and hip arthroplasty was selected from a population of 1339 patients. The patients were included in the study if they used the digital application for at least 6 weeks after surgery and completed their patient reported outcome measures preoperatively. Specifically, 82,189 days of activities were recorded and analyzed. Each patient provided written informed consent for the scientific use of their anonymized data.

### 2.2. Recording Device and Outcomes

Data collection occurred through the use of the moveUP^®^ application, a registered medical device based in Brussels, Belgium. Functioning as a smart virtual platform, this application is tailored for digital monitoring and incorporates a combination of both objective and subjective patient data. Comprising a patient-facing mobile application and a web-based dashboard, it serves as a comprehensive tool for both patients and healthcare providers. The database comprises data from patients who underwent hip and knee arthroplasty across Belgium, France, and the Netherlands. This application operates on a smart virtual platform designed for digital monitoring, utilizing both objective and subjective patient data. The platform consists of two main components: a patient-facing mobile application and a web-based dashboard utilized by the care provider. The objective data were collected using a commercial activity tracker (Garmin Vivofit 4 (Garmin Ltd., Olathe, KS, USA)) worn 24/7 by the patients. This device has demonstrated accuracy in recording step counts for older adults, particularly at elevated walking speeds and during outdoor walking activities [28]. It is noteworthy, however, that the device tends to underestimate step counts, especially at slower walking speeds and when individuals are engaged in indoor walking with frequent postural transitions [29]. The selection of the Garmin Vivofit^®^4 for our data collection process was informed by its recognized reliability in capturing physical activity metrics, aligning with the specific considerations highlighted in the literature [30].

The objective data consisted of the number of steps per day and the number of steps per minute throughout the day. Those data were collected during the rehabilitation period for which frequent interactions are occurring between the patient and care providers (Time in system—Table 1). The clinical data and patient-reported outcomes were also measured outside of the rehabilitation period. The patient-reported outcomes included the Oxford Knee Score, Forgotten Joint Score (FJS), Hip Osteoarthritis Outcome score (KOOS), Knee Osteoarthritis Outcome score (KOOS), UCLA Activity Scale (UCLA), and the EuroQol 5-Dimension (EQ5D).

### 2.3. Procedure

The preoperative parameters were first explored, as well as the impact of the demographics of surgery type. Then we explored the variability of the activity parameters throughout the recovery process by assessing the intraweek variability.

**Figure 1:**
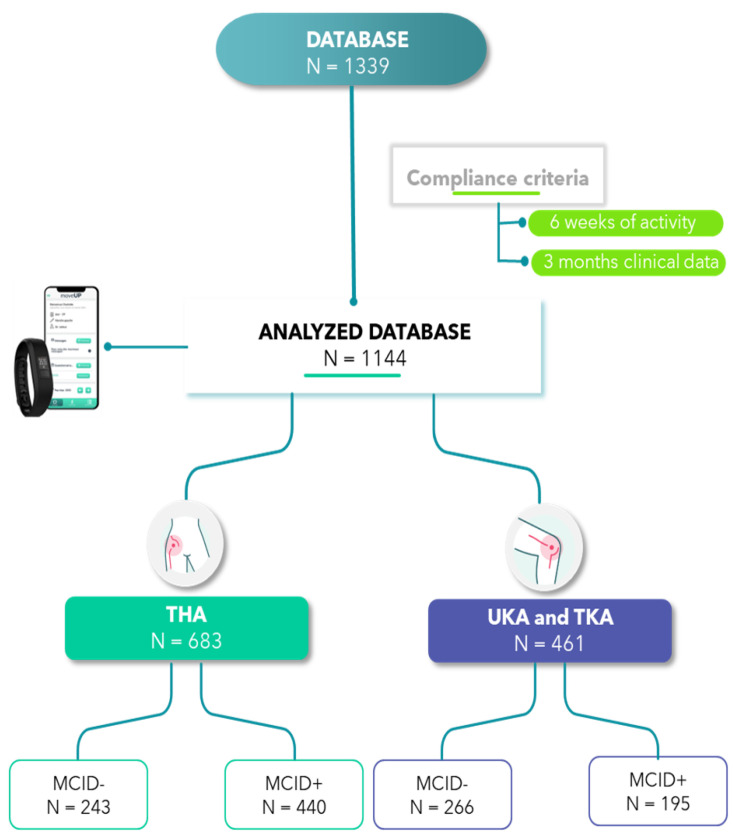
Data flowchart, note that for knee, the threshold use to determine MCID+ and − are different for UKA and TKA [31,32].

To explore the impact of quick or slow recovery trajectories on activity data, we used the JFS Minimal Clinically Important Difference (MCID) as a threshold at 3 months to divide the hip and knee patients into two groups: MCID achieved (MCID+) and MCID non-achieved (MCID−). The thresholds for MCIDs are 17.5 for THA [31], 16.6 for TKA [32], and 12.5 for unicondylar [31]. The flowchart on patient selection is presented in Figure 1. 

Finally, we investigated the impact of the type of knee surgery on activity metrics.

### 2.4. Data Processing

The Garmin activity tracker allows the user to collect the total number of steps each day, but the focal point of the data collection was the step-per-minute data [33]. This continuous stream of data was automatically stored in the moveUP mobile application via an SDK connection, providing real-time access to the participants’ dynamic activity profiles. To derive meaningful insights from the step-per-minute data, two key parameters were extracted: Peak 1-minute cadence (P1M) and Peak 6-minute consecutive cadence (P6MC). Those metrics were computed for each day of recording.

**Table 1:** Variable definitions.

Variable	Definition
Steps	Total steps accumulated in a day
P1M, cadence	Steps/minute recorded for the highest minute in a day
P6MC, cadence	Steps/6 min recorded for 6 consecutive minutes in a day
Light intensity, minute per week	Total number of minutes at <100 steps/minute
Moderate intensity, minute per week	Total number of minutes at >100 and <130 steps/minute
Vigorous intensity, minute per week	Total number of minutes at >130 steps/minute

#### 2.4.1. Peak 1-Minute Cadence (P1M)

The Garmin activity tracker allowed for the identification of the highest step count within any given minute throughout the day [34,35]. This metric, termed the P1M, served as an indicator of the participants’ maximal exertion or bursts of activity during their daily routines. Peak 1-min may represent one’s ‘best natural effort’, or rather, the free-living walking cadence of which an individual is capable. Peak-1 min cadence is highly dependent on age, physical activity level (i.e., steps/day), physical function, and body mass index (BMI) [33,36,37].

#### 2.4.2. Peak 6-Minute Consecutive Cadence (P6MC)

The highest continuous activity during 6 min is detected in step data, using a sliding 6 min window with 1 min overlap [38]. The one with the largest number of steps is chosen as the most representative in order to obtain the highest intensity reached during 6 consecutives minutes.

(1) $${P6MC}_{daily}=max(\frac{\sum_{i=j}^{6}X_{i}}{6})$$

With the first minute of day ≤ *j* ≤ last minute of day—6.

#### 2.4.3. Intensity

Walking cadence is a valid proxy of physical activity intensity. Moderate intensity is defined as activity above 3 metabolic equivalents (METs), which corresponds to a threshold of 100 steps/min [39]. Light intensity physical activity is defined as activity between 1.6 and 2.9 METs [40]. Activities under 20 steps/min are considered as incidental movements and are considered sedentary behavior [37]. The results were presented as the minutes spent per week to be consistent with the World Health Organization recommendation about the minimal level of physical activity [41].

#### 2.4.4. Outlier Removal

To ensure the integrity and accuracy of the step-per-minute data, a rigorous outlier removal process was implemented. Instances where step-per-minute data exceeded 150 were considered outliers, as such values were deemed unrealistic for the targeted population undergoing knee and hip arthroplasty rehabilitation. These outliers were systematically identified and removed from the dataset to prevent skewing of results and to maintain the reliability of the recorded activity metrics.

### 2.5. Statistical Analysis

The normality of each parameter was checked using graphical methods (boxplots, histograms and Q–Q plots). The data were presented as mean (standard deviation) or median [p25; p75] according to the type of distribution.

To determine the most stable indicators of the gross motor function of the patients, we assessed the intraweek variability of the parameters using the coefficient of variation across the seven days of the week.

We analyzed the different outcomes of both knee and hip OA patients using mixed models [42]. For each outcome, a mixed model with random intercept was used. The values from each day were treated as repeated measures. The model equation is

(2) $${Outcome}_{i,t}=\beta_{1}{day}_{t}+\beta_{2}{recovery}_{t}+\beta_{3}{\left(day\times recovery\right)}_{t}+\beta_{4}age+\beta_{5}gender\varepsilon_{i,t}+(\alpha +\alpha_{i})$$

With

(3) $$\varepsilon_{i,t}\sim (0,\sigma^{2})$$

(4) $$\alpha_{i}\sim (0,\mu^{2})$$

where α and $\beta_{1}$, $\beta_{2}$, and $\beta_{3}$ were employed as fixed effects, while $\varepsilon_{i,t}$ was used to represent random errors. The parameter $\alpha_{i}$ was utilized to quantify the random effect. The values of the parameters α, $\alpha_{i}$, $\beta_{1}:\beta_{5}$, and $\varepsilon_{i,t}$ were determined using maximum likelihood estimation (MLE) in the mixed-effects models.

Our analysis incorporated fixed effects related to recovery, days after surgery (spanning from 1 to 60 for hip and 1 to 90 for knee surgeries), as well as the interaction between the two. To ensure comparability, we imposed constraints on the estimated baseline measures. This was achieved by normalizing all groups and subtracting the mean values of each group’s first session from all subsequent sessions. Essentially, this constraint allowed for the adjustment of baseline measurements and accommodated variations in the relationship between baseline and follow-up scores across different sessions.

To assess the adequacy of the mixed-effects models, the following underlying assumptions were checked. QQ-Plot and boxplots were used to check the normality of the residuals. To assess the homoscedasticity of the residuals, we plotted residuals against predicted values. Finally, we examined linearity by evaluating the correlation between the predictors and the outcome.

It is important to note that centering the explanatory variables using mean values facilitated the direct interpretation of this effect as an intergroup effect [43].

We then computed the time, and the associated 95% confidence intervals, needed to differentiate between the recovery status.

The statistical analyses were performed at an overall significance level of 0.05. The statistical analyses were conducted in RStudio (version 2023.09.0) with R version 4.4.2., using the LME4 package to run the mixed-effect models [44].

## 3. Results

The details of the patient population are displayed in Table 2.

Significant differences were observed between the hip and knee population for the age, body mass index (BMI), patients reported outcomes, and the time of using the recording system. The age difference was 1.4 years [95%CI −2.43; −0.26], the patients undergoing hip surgery being younger than those for total knee replacement.

### 3.1. Preoperative Scores

First, we analyzed the data from the preoperative evaluation. Interestingly, when analyzing the overall number of steps per day, we did not find any statistically significant difference between hip and knee OA patients. However, when using the P6MC and the P1M, we did find statistically significant differences with higher cadences in patients with hip OA. The complete results are presented in Table 3.

**Figure 2:**
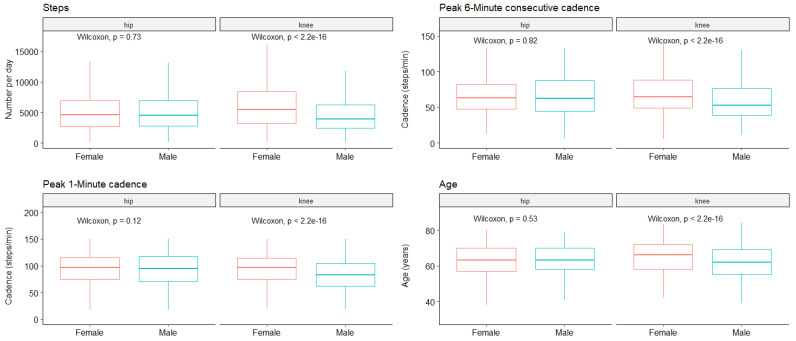
Preoperative characteristics according to gender and localization.

Next, to determine whether a separate analysis is necessary for female and male patients, we compared the pre-operative data based on gender (Figure 2).

On the one hand, for hip OA, we did not find statistically significant differences for the P1M (97 [75–115] and 95 [71–117] for female and male, respectively, *p* = 0.12), the P6MC (63 [48–83] and 62 [45–88], *p* = 0.81), or for the total number of steps per day (4628 [2673–6920] and 4508 [2814–6939], *p* = 0.73), nor for age (62.7 (8.9) and 62.6 (9.4), *p* = 0.83).

On the other hand, for knee OA, statistically significant differences were found for the total number of steps (5453 [3190–8444] and 3917 [2432–6217] for female and male, respectively, *p* < 0.001), the P6MC (65 [49–88] and 53 [39–76], *p* < 0.001), and the P1M (97 [75–115] and 83 [62–105], *p* < 0.001). Interestingly we found a statistically significant difference in the age at surgery, women being older than men (65.1 (8.5) and 61.7 (8.3), *p* < 0.001).

**Figure 3:**
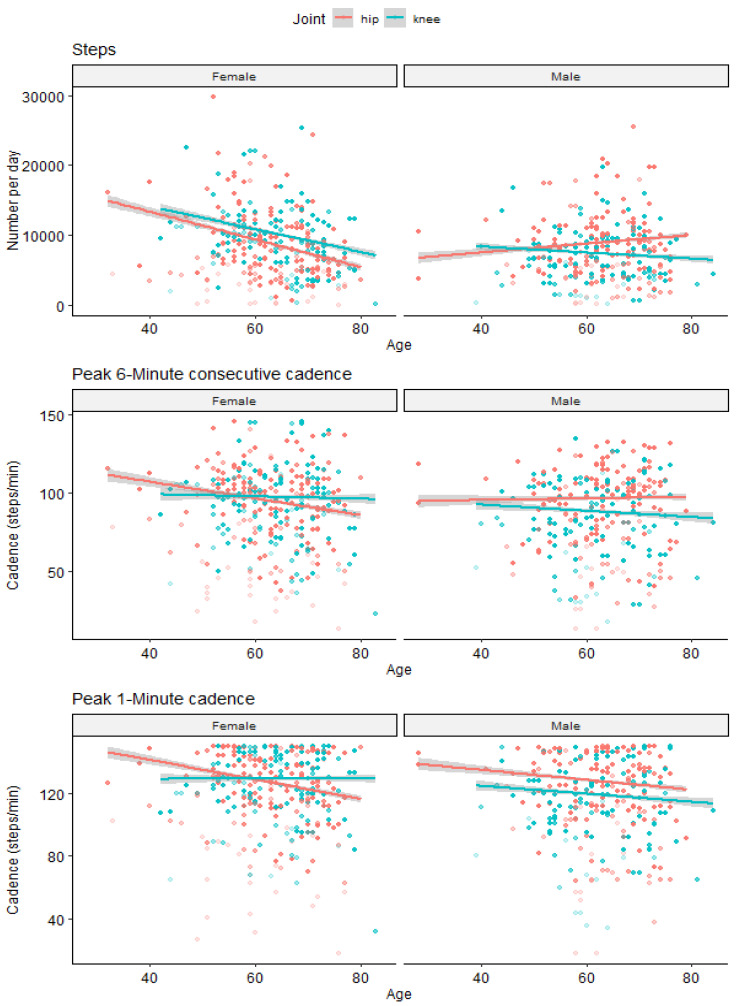
Evolution of the preoperative parameters according to age of the patients. The grey bands represent the 95% confidence intervals.

To further evaluate the potential influence of the age difference observed in knee patients, we performed linear regression to determine if age has an influence on the different studied parameters. The scatter plots are presented in Figure 3.

For female participants, concerning the total number of steps per day, the interaction between age and joint is not significant (*p* = 0.07), but there is a significant effect of age (β = −86 (8), *p* < 0.001) and joint (β = 1000 (141), *p* < 0.001). For the P6MC, there is no interaction between age and OA location (*p* = 0.23), but there is a significant effect of both age (β = −0.47 (0.05), *p* < 0.001) and joint (β = 3.2 (0.9), *p* = 0.001). For the P1M, there is a significant interaction between age and joint (β = 0.26 (0.12), *p* = 0.031), as well as a significant effect of age (β = −0.54 (0.07), *p* < 0.001) and joint (−16 (8), *p* = 0.042).

For male participants, concerning the total number of steps per day the interaction between age and joint is not significant (*p* = 0.44), and there is no statistically significant effect of age (β = 0.33 (0.61), *p* = 0.96) but a significant effect of joint (β = −720 (120), *p* < 0.001). For the P6MC, there is no interaction between age and joint (*p* = 0.79), nor a significant effect of age (β = −0.01 (0.05), *p* = 0.85), but a significant effect of joint (β = −8.3 (0.9), *p* < 0.001). For the P1M, there is no interaction between age and joint (*p* = 0.09) but significant effects of age (β = −0.02 (0.06), *p* < 0.001) and joint (−9.8 (1.1), *p* < 0.001).

### 3.2. Variability of the Outcomes

**Figure 4:**
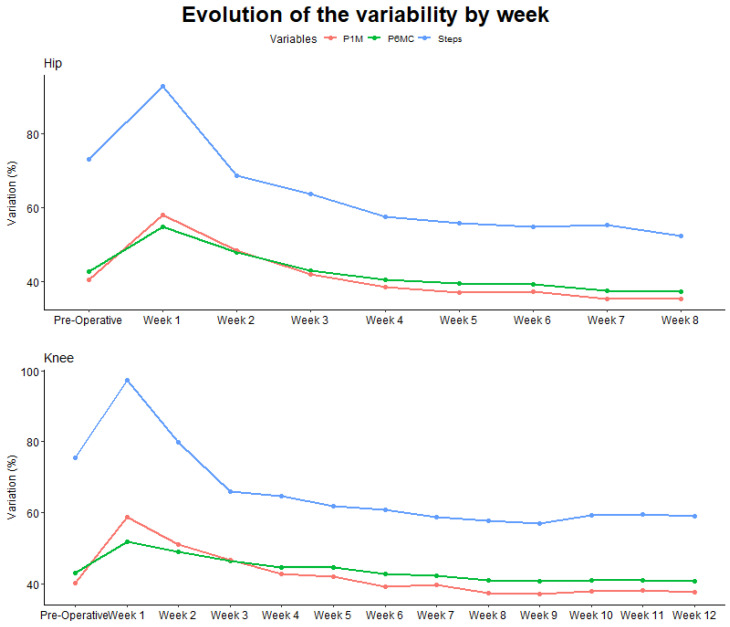
Evolution of the intraweek variability of the different studied parameters.

As presented in Figure 4, we can clearly see a difference in terms of variability between, on the one hand, the total number of steps per day, presenting a lot of variability, and on the other hand, the cadence during the P6MC and the P1M being much more consistent through the day.

### 3.3. Evolution of the Parameters during the Rehabilitation Process

#### 3.3.1. According to Recovery

**Figure 5:**
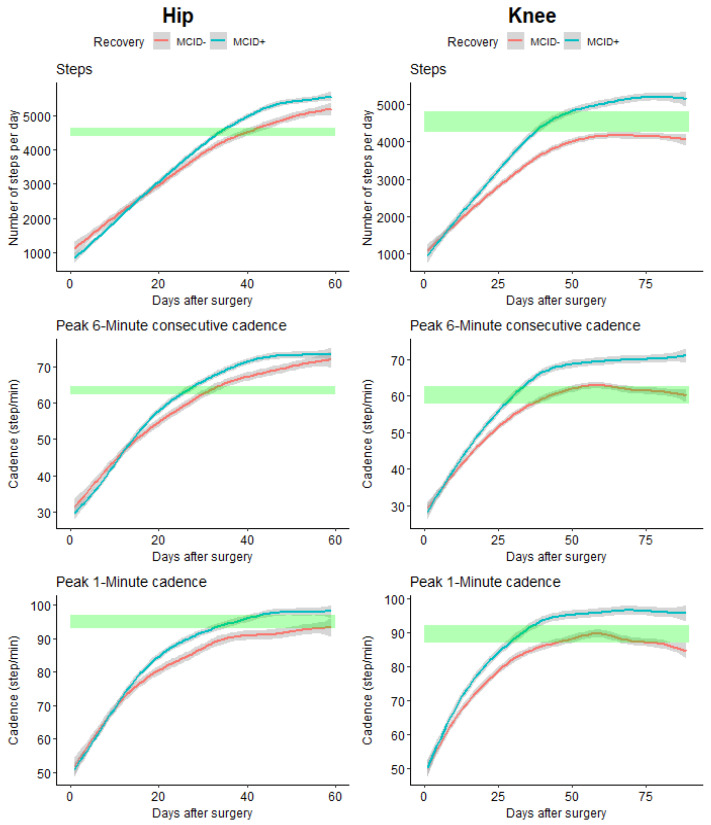
Evolution of the different studied parameters for hip and knee according to recovery. The green rectangles indicate the pre-operative values (with 95% CI).

When comparing the time needed to return to initial (pre-operative) values, important differences were observed for the computed outcomes (see Figure 5). 

THA patients who did not reach their MCID at 3 months (MCID−) took statistically 7 days more to recover their pre-operative activity level (number of steps) than patients who reached their MCID at 3 months (MCID+) (33 days for MCID+ and 40 days for MCID−); for the P6MC, a difference of 6 days (26 days for MCID+ and 32 days for MCID−); for P1M, the MCID+ regains the initial value after 35 days, while the MCID− did not reach the initial value after the 60 days of follow-up.

For TKA, for the number of steps, only the MCID+ group reached the initial value after 40 days; for the P6MC, a difference of 10 days (29 days for MCID+ and 39 days for MCID−); and for P1M, a difference of 12 days (38 days for MCID+ and 50 days for MCID−).

When comparing the trajectory of the evolution in the different groups, we observed that for both THA and TKA, the newly developed outcomes, P6MC and P1M, allowed for early identification of differences in comparison with the total number of steps per day (Table 4).

**Figure 6:**
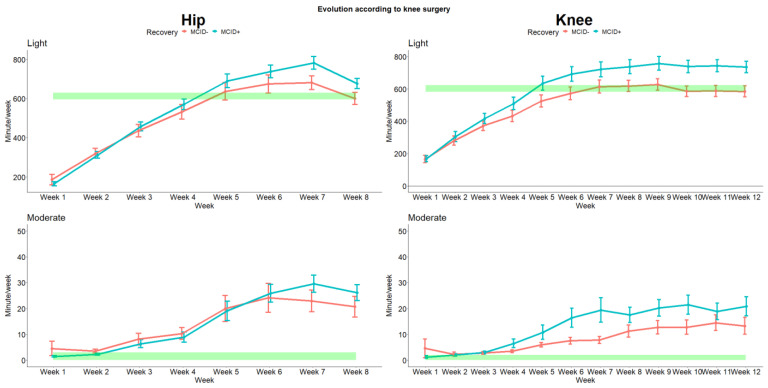
Evolution of the level of intensity for hip and knee according to recovery. The green rectangles indicate the pre-operative values (with 95% CI).

Then, we analyzed the intensity of the activities through the rehabilitation process. The evolution for light and moderate activities are presented in Figure 6 (due to the quasi absence of vigorous activities, these results were not presented). As for the number of steps and cadence, we observed differences between the recovery status. Interestingly, we also observed that both THA and TKA patients are quickly able to recover higher levels of moderate activities that were barely absent pre-surgery.

#### 3.3.2. According to the Type of Surgery

For patients with knee prostheses, we were able to perform comparisons according to the type of surgery, which is an important point given the different clinics and symptomatologies of these patients. There was no statistically significant difference in age between both interventions (63.7 (6.2) and 62.7 (7.4) for TKR and UKA intervention, respectively, *p* = 0.44) and equal gender distribution (χ^2^ = 0.71, *p* = 0.70).

The results of the mixed-effect models are presented in Table 5 and Figure 6.

**Figure 7:**
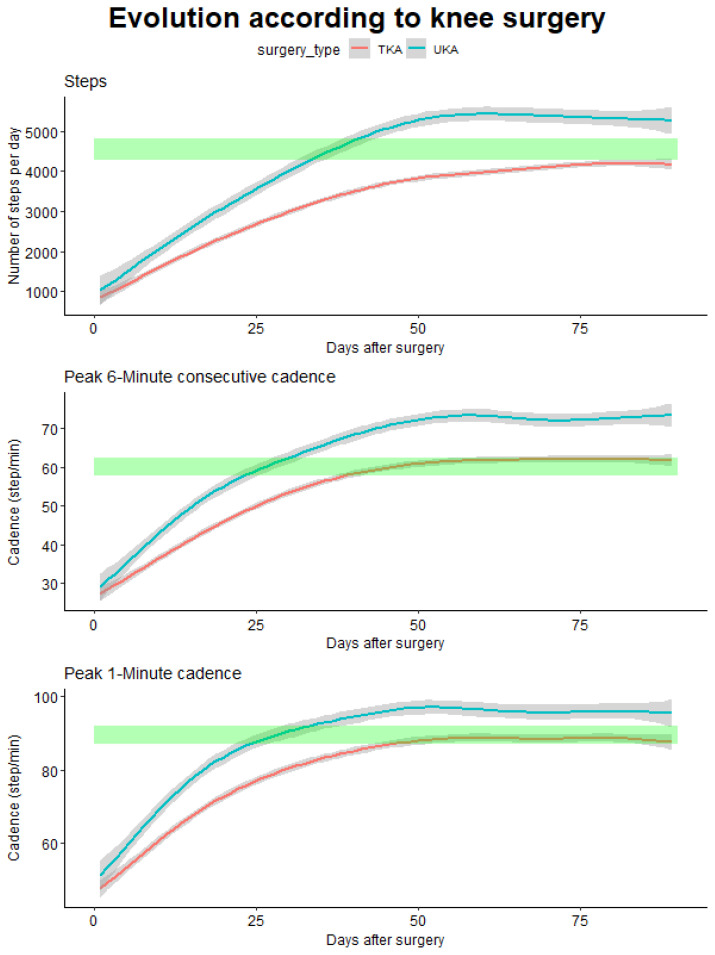
Evolution of the different studied parameters according to the type of knee prosthesis. The green rectangles indicate the pre-operative values (with 95% CI).

When comparing the time needed to return to pre-operative values, important differences were observed for the computed outcomes (Figure 7).

For the total number of steps, only patients with UKA surgery return to their initial values after 36 days and then continue to progress above pre-operative values, while patients with TKR barely return to their initial value after the 90 days. On the other hand, for the P6MC, a difference of 16 days was observed (25 days for UKA and 41 days for TKR), as well as for the P1M, where a difference of 23 days was observed (28 days for UKA and 51 days for TKR).

Again, when comparing the different outcomes to differentiate the two interventions, we observed that early differences are detected using the P6MC and P1M in comparison with the total number of steps.

## 4. Discussion

### 4.1. Main Results

The results of this study provide insights into the preoperative functional characteristics, variability of outcomes, and the evolution of activity parameters through the rehabilitation process in patients with hip and knee OA undergoing THA and TKA.

The initial noteworthy difference observed in preoperative parameters was the capacity of P6MC and P1M to distinguish between populations with knee and hip OA conditions. In contrast, the number of steps did not exhibit such discriminatory capacity. The gender-based analysis also revealed interesting nuances in preoperative characteristics. While no significant differences were observed in hip OA patients based on gender, significant differences were found in knee OA patients, including the total number of steps, P6MC cadence, P1M cadence, and age at surgery. Female knee OA patients exhibited higher values in these parameters. For both female and male patients, age and OA location showed significant effects on the total number of steps per day, P6MC cadence, and P1M cadence. These findings underscore the importance of considering age as a contributing factor in patients with hip and knee OA.

Intraweek variability analysis revealed notable differences among the studied parameters. While it is established that daily step count is correlated with cadence metrics [36], our study revealed that the variability in the number of steps is twice as substantial as that observed in cadence metrics. This suggests that cadence measurements such as P6MC and P1M may offer more stable indicators of gross motor function compared to the total number of steps per day.

Concerning the time required to return to preoperative values based on the type of arthroplasty (THA or TKA), significant differences were observed in the recovery trajectories, with the cadence metrics (P6MC and P1M) showing early identification of differences compared to the total number of steps per day. These results highlighted the potential of P6MC and P1M as sensitive measures for assessing the rehabilitation progress in both THA and TKA patients. The median value for P1M was similar to the normative value identified in older adults (106 ± 16 and 97 ± 20 for male and female, respectively [45]. Concerning the number of steps, our results aligned with patterns observed in numerous prior studies [46]. Patients typically resumed their pre-operative activity levels approximately six to ten weeks after undergoing surgery, which is consistent with findings from previous accelerometry investigations [21,47]. Notably, patients who underwent TKA experienced a slower recovery compared to those who received partial knee arthroplasty. This outcome mirrors the trends observed in the existing literature. Additionally, patients who underwent THA tended to recover gait quality more rapidly and demonstrated greater improvements compared to their pre-operative levels in line with previous studies, although most of these studies primarily focus on total step counts rather than walking session durations or patterns of activity accumulation in these patient populations [48,49].

The cadence is often regarded as a reasonable proxy-indicator of ambulatory intensity, with a cadence value of ≥100 steps/min in adults consistently identified as a heuristic for ‘good walking’ [37,50]. The newly introduced metric, P6MC, as compared to P1M and P30, exhibits an intriguing difference, wherein P6MC values are 20 to 30 steps lower than P1M, highlighting the distinction between these metrics. Considering established cadence thresholds: 40–59 for purposeful stepping, 60–79 for slow walking, 80–99 for medium walking, 100–119 for brisk walking, and >120 for fast walking [37]; it becomes apparent that our OA population only demonstrated recovery in slow walking during the 6-min duration, while brisk walking was restored within one minute. This could still be construed as a diminished functional capacity during the period assessed in this study.

The previous studies showed good correlation between P6MC cadence and the official P6MC distance; however, the direct transformation from cadence to distance is still challenging [38]. The sensitivity of this outcome in detecting older individuals with functional capacity has been demonstrated [51]. A gait cadence of 107 steps/min during a 6MWT has been identified as the threshold to distinguish older adults with an inability to walk, a minimum functional threshold (defined as 370 m) with reasonably high sensitivity (80%) and modest specificity (57%). Interestingly, it has also been shown that the unsupervised version of the 6-min walk test (random walk) gives the same results as the official (supervised) 6-min walk test [52].

The utilization of wearable sensors for assessing knee arthroplasty procedures is becoming increasingly prevalent [53]. However, the discernible clinical value of this technology is still a subject of ongoing investigation. One potential indicator under scrutiny is the variability in physical activity following surgery. Notably, patients exhibiting excessive variation in physical activity have been shown to experience more pronounced postoperative pain [54].

It is noteworthy that only a limited number of patients with knee or hip OA adhere to the physical activity guidelines set by the WHO [55]. In the context of TKA and THA, the previous studies indicated a rise in light activity at 6 months postoperatively, with no concomitant change in moderate or vigorous intensity [48]. In a comparative study the P1M improved from 70.0 ± 23.7 preoperatively to 91.5 ± 26 at 1 year postoperatively after TKA [56]. Our investigation suggests that this improvement is realized much earlier than the conventional one-year postoperative timeframe. The exploration of variables related to physical activity holds significance, given that heightened physical activity has been associated with improvements in gait function after TKA. A suggested cutoff point of 3053 steps per day emerged as a potentially valuable predictive factor for gait function following TKA [57]. It is to note that we observed a slight decrease in the level of physical activities, both light and moderate, after Week 8. This can be interpreted in the context of postoperative recovery among hip and knee patients. Notably, the hip patients engaged with the system for an average of 59 days post-surgery (Range: 50–69), while knee patients utilized it for an average of 78 days (Range: 50–91). Week 8 initiates at day 56 post-surgery, suggesting that patients who recovered well likely ceased using the system around this time. This temporal correlation suggests that the decline in activity minutes during Week 8 could be indicative of less intense activities undertaken by patients who are in the process of recovery. It is plausible that the observed drop in activity levels is reflective of the varying degrees of recovery among patients. Those who have recovered more robustly may transition to less intensive activities, contributing to the reduction in recorded minute/week values during this period.

The patients’ trajectories following arthroplasty display considerable variability, and extensive discussions have revolved around categorizing individuals as slow or quick progressors. Notably, these discussions have primarily centered on subjective questionnaires like patient-reported outcome measures [58]. The substantial variability in physical activity among patients has prompted calls for the development of stratification tools [59]. In our study, we categorized the patient population based on MCID of the FJS at the three-month mark. This time point aligns with routine medical consultations and, in some countries, marks the conclusion of bundled payments. Our analysis demonstrates that the activity data hold the potential to partially predict which patients will achieve this milestone early in the recovery process [60].

### 4.2. Strengths and Limitations

This study demonstrates several notable strengths that enhance its robustness and scientific significance. Foremost among these strengths is the study’s commitment to real-world relevance, evident in its recognition of the critical importance of assessing patients in ecological environment and conditions [61,62,63,64]. This approach stands in stark contrast to conventional clinical tests, providing a more ecologically valid perspective on the rehabilitation process.

A pivotal strength lies in the utilization of objective measurements facilitated by digital biomarkers collected through wearable sensors. This methodology not only ensures the continuous acquisition of real-time data but also offers a more nuanced and sensitive evaluation compared to subjective assessments by clinicians [65,66,67]. Furthermore, the integration of technology enables continuous monitoring between sessions, affording the ability to detect even subtle changes in a patient’s status [68,69,70]. This feature significantly enhances the precision of the rehabilitation process, ensuring a proactive response to evolving patient needs.

However, the results of this study also have to be seen with various limitations. First, we did not monitor wear time or adherence to the wearable devices. The evaluation of the acceptability of smartwatches and activity trackers typically centered around two primary factors: data availability and wearing time. While the majority of studies consistently reported high levels of both data availability and wearing time (>75%) [71], the events during the remaining 25% of the time remain unknown. This aspect could potentially influence the total number of steps, although its impact on cadence metrics may be less pronounced. Second, concerning the P6MC, previous studies have shown that this could be aggregated as an unsupervised 6 min walking test. However, we cannot be sure that the activity was walking [38,72]. However, the cumulative step count over the 6-min duration provides valuable information about participants’ endurance and functional capacity. Third, a notable study limitation is the potential variation in smartphone usage among participants. The study assumes a certain level of familiarity and consistent use of smartphones, which could introduce confounding factors if participants differ in their comfort and proficiency with such technology (i.e., digital literacy) [73]. Additionally, the success of the proposed approach relies on the widespread adoption and accessibility of smartphones and wearable sensors, which may not be universally available to all individuals undergoing rehabilitation [74]. This raises concerns about the generalizability of the study findings to diverse populations, potentially introducing biases based on socio-economic or demographic factors.

### 4.3. Future Works

As various factors influence activity recovery after surgery, such as the surgical approach [75], the use of crutches [47], and BMI [76]—it would be interesting to integrate these parameters in more complex model to better predict the evolution of the patients through the rehabilitation process.

The future research presents exciting opportunities for advancements and refinement of this methodology. First, the research into user engagement strategies with wearable devices could explore innovative approaches to enhance adherence without direct supervision. This could involve the development of user-friendly interfaces, personalized feedback systems, or even the incorporation of elements of gamification to promote sustained and consistent use.

Moreover, the future research could focus on expanding the scope of wearable technology beyond wearables, smartphones, exploring alternative devices or communication methods to cater to individuals who may not have access to wearables. This inclusivity-driven approach would contribute to a more comprehensive understanding of the benefits and challenges associated with wearables in diverse populations.

To bolster the validity and reliability of wearable sensors, the future research could delve into advancements in sensor technology, refining algorithms and conducting thorough validation studies. Comparative assessments against established clinical measures can provide a clearer understanding of the accuracy and potential limitations of these devices.

Additionally, exploring the integration of artificial intelligence and machine learning algorithms for data analysis could open new avenues for deriving meaningful insights from the vast amount of data collected through wearables. These technologies could help identify patterns, predict rehabilitation progress, and tailor interventions based on individual patient needs.

## 5. Conclusions

This study explores the capacity for technology-assisted rehabilitation to bring about significant changes, particularly through the incorporation of automated, unsupervised evaluations utilizing wearable sensors in the rehabilitation of patients who have undergone hip and knee replacement surgeries and suffer from OA. The study emphasizes the constraints of conventional clinical evaluations and the changing nature of real-life rehabilitation encounters. Using wearable sensors provides a patient-centered and ecologically sound method, addressing the limitations of subjective evaluations conducted in clinics.

The results highlight the importance of cadence measurements, the P6MC and the P1M, in differentiating between individuals with hip and knee conditions and monitoring their recovery progress. These indicators demonstrate sensitivity and surpass the total step count, offering useful insights into the functional capacity and progress of patients throughout rehabilitation.

The use of technology-based, self-directed evaluations represents a fundamental change in rehabilitation methods, offering improved accuracy, tailored treatments, and heightened patient involvement.

## Figures and Tables

**Table 2:** Clinical characteristics of the patients. Results are indicated s mean (std) or median [p25; p75] according to the distribution of the data.

Variables	Overall (*n =* 1144)	Hip (*n =* 683)	Knee (*n =* 461)	*p*-Value
Gender, female	580, 51%	348, 51%	232, 50%	0.30
Age, years	62 (10)	62 (10)	63 (10)	0.015
BMI, kg/m^2^	29.0 (10.6)	28.0 (11.1)	30.5 (9.7)	<0.001
Type of surgery				<0.001
*Total*	1010, 89%	665, 97%	345, 75%	
*Unicondylar*	101, 8.9%	/	101, 22%	
*Revision*	22, 1.9%	10, 1.5%	12, 2.6%	
*Resurfacing*	8, 0.7%	8, 1.2%	/	
Oxford Score	24 (8)	24 (8)	25 (8)	0.21
FJS	10 [4; 20]	10 [4; 21]	10 [4; 19]	0.19
OOS				
*Pain*	46 (17)	45 (18)	46 (16)	0.41
*Symptoms*	50 (18)	47 (18)	54 (17)	<0.001
*ADL*	48 (18)	46 (18)	50 (18)	<0.001
*QoL*	30 (18)	31 (19)	29 (16)	0.30
*Leisure and Sport*	19 [5; 31]	25 [6; 38]	10 [0; 25]	<0.001
UCLA	3 [2; 5]	3 [2; 5]	3 [2; 5]	0.30
Time in system, days	81 [63; 102]	76 [61; 96]	91 [65; 110]	<0.001
Time in system since intervention, days	62 [50; 85]	59 [50; 69]	78 [50; 91]	<0.001

FJS: Forgotten Joint Score, KOOS: Knee Injury and Osteoarthritis Outcome Score, HOOS: Knee Injury and Osteoarthritis Outcome Score, UCLA: UCLA Activity Scale.

**Table 3:** Pre-operative functional characteristics of the included patients; median [p25; p75].

Variables	Overall (*n =* 5806)	Hip (*n =* 3267)	Knee (*n =* 2539)	*p*-Value
Steps, n	4477 [2601; 6941]	4495 [2618; 6865]	4455 [2569; 7072]	0.90
P6MC, cadence	61 [44; 85]	63 [46; 85]	58 [43; 82]	<0.001
P1M, cadence	92 [68; 115]	95 [70; 118]	89 [66–110]	<0.001
Intensity, min/week				
*Light*	613 [371; 938]	619 [364; 910]	609 [315; 968]	<0.001
*Moderate*	0 [0; 14]	0 [0; 14]	0 [0; 7]	0.80
*Vigorous*	0 [0; 0]	0 [0; 0]	0 [0; 0]	0.89

*n* represents the number of days recorded.

**Table 4:** Results of the mixed effect model analysis, including the interaction with the recovery status and the days. β and standard errors are presented.

Variables	Day	Recovery	Age	Gender	Day × Recovery	Diff.
	Hip					
Steps, n	69.0 (1.1)	443 (77)	−11.8 (7.9)	−513 (155)	16.5 (1.3)	25
P6MC, cadence	0.65 (0.01)	2.44 (0.87)	−0.04 (0.08)	−4.34 (1.58)	0.10 (0.01)	16
P1M, cadence	0.62 (0.01)	2.7 (1.0)	−0.11 (0.8)	−4.1 (1.6)	0.13 (0.02)	15
	**Knee**					
Steps, n	36.5 (0.5)	452 (62)	−12 (10)	−720 (201)	11.8 (0.8)	14
P6MC, cadence	0.33 (0.01)	3.4 (0.7)	−0.03 (0.01)	−6.4 (2.0)	0.09 (0.01)	13
P1M, cadence	0.35 (0.01)	2.6 (0.8)	0.09 (0.10)	−6.8 (2.2)	0.08 (0.01)	9

Diff. Difference (days); for Recovery, the MCID+ is the reference; for Gender, the reference is the female group.

**Table 5:** Results of the mixed-effect model analysis for patients with TKR, including the interaction with the type of prosthesis and the days. β and standard errors are presented.

Variables	Knee			
	Day	Type	Day × Type	Diff.
Steps, n	50 (1)	392 (147)	11 (2)	7
P6MC, cadence	0.5 (0.01)	4.8 (2.5)	0.02 (0.02)	4
P1M, cadence	0.5 (0.01)	6.3 (3.2)	0.03 (0.02)	3

Diff. Difference (days).

## Data Availability

The data are unavailable due to privacy or ethical restrictions.
